# Creatine Monohydrate Supplementation Increases White Adipose Tissue Mitochondrial Markers in Male and Female Rats in a Depot Specific Manner

**DOI:** 10.3390/nu13072406

**Published:** 2021-07-14

**Authors:** Chantal R. Ryan, Michael S. Finch, Tyler C. Dunham, Jensen E. Murphy, Brian D. Roy, Rebecca E. K. MacPherson

**Affiliations:** 1 Department of Health Sciences, Brock University, St. Catharines, ON L2S 3A1, Canada; cr13ai@brocku.ca (C.R.R.); mf15kj@brocku.ca (M.S.F.); 2 Department of Kinesiology, Brock University, St. Catharines, ON L2S 3A1, Canada; td13nb@brocku.ca (T.C.D.); jm15ln@brocku.ca (J.E.M.); broy@brocku.ca (B.D.R.)

**Keywords:** adipose tissue, creatine, mitochondria, thermogenesis

## Abstract

White adipose tissue (WAT) is a dynamic endocrine organ that can play a significant role in thermoregulation. WAT has the capacity to adopt structural and functional characteristics of the more metabolically active brown adipose tissue (BAT) and contribute to non-shivering thermogenesis under specific stimuli. Non-shivering thermogenesis was previously thought to be uncoupling protein 1 (UCP1)-dependent however, recent evidence suggests that UCP1-independent mechanisms of thermogenesis exist. Namely, futile creatine cycling has been identified as a contributor to WAT thermogenesis. The purpose of this study was to examine the efficacy of creatine supplementation to alter mitochondrial markers as well as adipocyte size and multilocularity in inguinal (iWAT), gonadal (gWAT), and BAT. Thirty-two male and female Sprague-Dawley rats were treated with varying doses (0 g/L, 2.5 g/L, 5 g/L, and 10 g/L) of creatine monohydrate for 8 weeks. We demonstrate that mitochondrial markers respond in a sex and depot specific manner. In iWAT, female rats displayed significant increases in COXIV, PDH-E1alpha, and cytochrome C protein content. Male rats exhibited gWAT specific increases in COXIV and PDH-E1alpha protein content. This study supports creatine supplementation as a potential method of UCP1-independant thermogenesis and highlights the importance of taking a sex-specific approach when examining the efficacy of browning therapeutics in future research.

## 1. Introduction

The activation of white adipose tissue (WAT) into a more metabolically active tissue has become a burning topic in obesity prevention and treatment. Today, WAT is no longer regarded as an inert storage depot for triacylglycerides, but is considered to be a highly plastic endocrine organ that can undergo dramatic phenotypic changes in response to different stresses (i.e., exercise, cold) [1,2,3,4]. Recently, there has been a large focus on the mechanisms by which WAT can be recruited to resemble the phenotypical and functional characteristics of the more metabolically active brown adipose tissue (BAT). This process is known as adipose tissue “browning” and results in the activation of beige adipocytes that reside in WAT depots [5]. Lineage tracing and molecular analysis indicates these beige adipocytes are distinct thermogenic fat cells [6], however beige adipocytes can convert to a WAT phenotype when the stimulus, such as cold exposure, is terminated [7], indicating that further work is necessary to fully distinguish white and beige adipocyte lineages. Considering the vast amount of WAT that individuals possess, the activation of WAT into the more metabolically active form of beige adipose tissue has the potential to significantly increase daily energy expenditure [4,8]. Thus, it could be regarded as a potential therapeutic treatment to combat the obesity epidemic and its related comorbidities.

Morphologically, mature differentiated white adipocytes are described as unilocular—containing a single large lipid droplet, few mitochondria, and a nucleus that has been pushed to the border of the cell membrane [9]. In contrast, brown adipose tissue (BAT) is described as multilocular with several smaller lipid droplets, a more central nucleus, and a dense population of mitochondria, ultimately giving the tissue its characteristic brown appearance [9]. Beige adipocytes lie along a continuum between WAT and BAT. The thermogenic capacity of brown and beige fat relies predominantly on a fatty acid/H+ symport mechanism mediated by uncoupling protein 1 (UCP1) [10,11,12,13]. This inner mitochondrial membrane protein stimulates thermogenesis by uncoupling the electron transport chain. Specifically, UCP1 dissipates the proton motive force and increases the rate of substrate flux through the mitochondrial electron transport chain [14]. This UCP1 dependent process is the most studied mechanism underlying the thermogenic capabilities of BAT, however, recently, UCP1-independent modes of thermogenesis have been uncovered.

In comparing the response of wildtype and UCP1 knockout mice to adrenergic stimulation, it was found that the thermogenic response (pharmacological and cold-induced) was similar [15,16,17,18], implying the presence of UCP1-independent thermogenic mechanisms. Previous work has found that reductions in high energy-phosphate compounds, such as creatine, result in a dysregulation of thermogenesis [19,20] and a role for creatine cycling in adipose tissue thermogenesis was suggested almost four decades ago [1]. In 2015, Kazak et al. [14] performed quantitative mitochondrial proteomics and identified creatine metabolism as a signature of beige fat from cold-exposed mice. Interestingly, both genetic and pharmacological depletion of adipose creatine potentiates diet-induced obesity [21,22], and inactivation of creatine transport results in fat accumulation [21]. In adipose tissue, the creatine pool is regulated by intracellular synthesis and by influx from circulation. The forward and reverse phospho-transfer reactions of phosphocreatine (PCr)/creatine in most cells occur in a 1:1 stoichiometry with the ATP/ADP coupled. However, in thermogenic adipocytes, it has been estimated that there is an excessive release of ADP with respect to creatine [14,23]. Therefore, it is thought that creatine facilitates the regeneration of ADP through futile hydrolysis of PCr [14]. Importantly, it was recently demonstrated that creatine kinase B (CKB) traffics to the mitochondria where it plays an important role in the futile creatine cycle [24] and that tissue-nonspecific alkaline phosphatase (TNAP) localizes to the mitochondria where it acts as a robust PCr phosphatase in thermogenic fat [25]. Together, these studies demonstrate that adipocyte creatine energetics can be a key regulator of thermogenesis [14,20,21,22,23].

Creatine monohydrate (CM) is a stable form of creatine with an attached molecule of water and is one of the most widely used and researched oral supplements (for review [26,27]. CM supplementation is well known for its effects on enhancing body composition, muscle mass and health, and exercise performance [28,29]. Supplementation is commonly in the range of 5 to 20 g/day which all lie within a range that is safe and tolerable for consumption [27,30]. While most of the work in the area has focused on CM supplementation and skeletal muscle outcomes, limited investigations have been performed to demonstrate that CM supplementation can increase creatine concentrations in other tissues. CM supplementation increases creatine concentrations in cardiac muscle, brain, kidney, liver, and lung tissue female rodents [31]—however, adipose tissue was not analyzed.

The purpose of the current investigation was to determine the effect of creatine supplementation on WAT browning in male and female Sprague-Dawley rats. To examine this question, male and female rats were separated into four different groups and supplemented with different doses of creatine (0, 2.5, 5 and 10 g/L) for 8 weeks. The results of this study provide novel information about the potential of creatine supplementation to induce WAT browning and further provide insight into the sex-specific responses of creatine supplementation.

## 2. Materials and Methods

### 2.1. Animals and Study Design

Experimental protocols were approved by the Brock University Animal Care Committee (file #19-02-01) and are in compliance with the Canadian Council on Animal Care. Thirty-two Sprague-Dawley rats (16 male, average body weight 372.4 g ± 12.1 g; 16 female, average body weight 311.8 g ± 21.0 g) were ordered from Charles River Laboratories (Wilmington, MA, USA) at 11 weeks of age. Rats acclimatized for 7 days in the Brock University Comparative Biosciences Facility. All rats were kept on a 12-h light:12-h dark cycle and had ad libitum access to food (AIN-93G pellets) and water throughout the duration of the study. Rats were separated by sex, housed in pairs, and randomly assigned into one of four experimental groups: (1) control (1% sucrose via drinking water), (2) 2.5 g/L, (3) 5 g/L, and (4) 10 g/L of creatine monohydrate (CM) (Sigma-Aldrich; CAT# C3630) and 1% sucrose via drinking water. Incremental doses of creatine monohydrate were selected to determine if there would be a dose response in adipose tissue adaptations. Rat weight, food, and water intake were measured three times a week for 8 weeks [32].

### 2.2. Tissue Collection and Homogenization

After the 8-week feeding period, rats underwent non-survival surgeries using isoflurane gas anesthesia. WAT samples were collected from inguinal subcutaneous fat depots (iWAT) and gonadal fat depots (gWAT; epididymal and ovarian), while BAT samples were collected from the interscapular fat pads. Samples were divided and placed in either formalin for histological analysis or snap frozen in liquid nitrogen and stored at −80 °C for analysis via Western blotting. Blood samples were collected from the heart and rats were euthanized via exsanguination.

### 2.3. Western Blotting

Samples were homogenized via FAST prep (FastPrep^®^, MP Biomedicals, Santa Ana, CA, USA) in 3 and 10 volumes (WAT and BAT samples respectively) of NP40 Cell Lysis Buffer (Life Technologies; CAT# FNN0021) supplemented with 34 µL phenylmethylsulfonyl fluoride and 50 µL protease inhibitor cocktail (Sigma; CAT# 7626-5G, CAT# P274-1BIL). Homogenates were then centrifuged at 4 °C for 5 min at 1500× *g*, after which the infranatant was collected and protein concentration was determined using a Bicinchoninic acid assay (Sigma-Aldrich—B9643, VWR—BDH9312). Homogenates were prepared in 2× Laemmli sample buffer (1 µg/µL) and were denatured at 100 °C for 5 min. Equal amounts of sample (10–20 µg) were loaded to undergo protein separation via SDS-PAGE (4% stacking, 10–15% resolving gel) for 90 min at 120 V). Protein was wet transferred to 0.45 µm nitrocellulose membranes (CAT# 10600001, Milipore Sigma Burlington, MA, USA) at 100 V for 60 min. Membranes were blocked in tris buffered saline/0.1% tween 20 (TBST) prepared with 5% non-fat dry milk for 1 h followed by overnight incubation at 4 °C with the appropriate primary antibody. Following primary incubation, membranes were rinsed with TBST and incubated with the appropriate Horseradish peroxidase-conjugated secondary antibodies for 1 h at room temperature. Ponceau staining was used to confirm equal protein loading (<10% variability across the membrane). Signals were detected using enhanced chemiluminescence (Western Lightening ^®^ Plus ECL, Perkin Elmer, MA, USA) and were subsequently quantified by densitometry using a FluorChem HD imaging system (Alpha Innotech, Santa Clara, CA, USA). The primary antibodies included: cytochrome C (ABCAM, CAT# ab76237), citrate synthase (ABCAM, CAT# ab96600), COXIV (Molecular Probes, CAT# A-21348), PDH (Millipore, CAT# ABS2082), UCP-1 (ABCAM, CAT# ab10983), PGC-1α (Millipore, CAT# AB3242), GAMT (ABCAM, CAT# ab126736), and CKB (Abclonal; cat. no. ab12631).

### 2.4. Real-Time qPCR

Adipose mRNA was extracted and reverse transcribed into cDNA and changes in mRNA expression were determined using real-time quantitative PCR as described previously [33]. RNA was isolated from adipose tissue following homogenization in Trizol reagent using an RNeasy kit according to the manufacturer’s instructions (RNeasy Kit 74106; Qiagen). RNA yield and purity were determined using a Nano-drop system (NanoVue plus; GE healthcare). RNA samples were prepared at 1 μg/μL using RNase free water. cDNA was synthesized using random primers and dNTP (Invitrogen) at a 1:1 ratio as well as a master mix (5 × FSB, DTT, RNase out and SuperScript II Reverse Transcriptase). 7500 Fast Real-Time PCR system (Applied Biosystems) was used to perform the RT qPCR. Samples were loaded in duplicate and contained 10 μL of PCR master mix, 4 μL of RNase free water, 1 μL of gene expression assay, and 5 μL of cDNA. Gene expression assays were purchased for *Slc6a8* (Rn00506029_mL). *Gapdh* was used as a housekeeping gene and was not different between groups. Relative differences in *Slc6a8* were determined using the 2^-∆∆CT^ method and normalized to the respective control group [34].

### 2.5. Histology

Samples (gWAT, iWAT, and BAT) underwent fixation in 10% neutral buffered formalin (Millipore Sigma, CAT#HT501128) for 62 h. Following fixation, samples were transferred into 70% ethanol for future processing. Samples underwent dehydration via ethanol (1 × 90% 30 min, 3 × 100% 40 min) and xylene (Fischer Scientific) (3 × 45 min). Samples were embedded in paraffin and 10 µm sections were mounted on 1.2 mm Superfrost™ slides. Slides were stained with Harris hematoxylin and eosin (H&E), imaged using a Nikon Eclipse 80i microscope (CAT#PL-D655CU-CYL), and images were captured with Pixelink software. Three images from each animal (~150 cells/image) were sampled to determine cross-sectional area and percent multilocular (ImageJ software, National Institute of Mental Health, Bethesda, MD, USA).

### 2.6. Statistical Analysis

Control male and female comparisons were made with a one-way analysis of variance (ANOVA). Comparisons within sex and across doses were made with a one-way ANOVA with all measurements being made relative to the control groups. Post-hoc analysis was completed with a Tukey’s multiple comparisons test. Statistical significance was assumed at *p* ≤ 0.05, and GraphPad Prism 8 software (GraphPad Software, La Jolla, CA, USA) was used to perform all statistical analyses. Results are stated and presented as mean ± SEM for all groups.

## 3. Results

### 3.1. Animal and Adipose Tissue Depot Characteristics

No differences were observed for male or female rat body mass at the end of the intervention between creatine doses. Female rats had a lower body mass compared to males in each creatine supplementation group (*p* < 0.05). Food and water intake were not different for either male or female rats in each creatine supplementation group. Normalizing for body mass, female rats ate more when compared to males in each treatment group (*p* < 0.05). Total creatine consumption relative to body mass was different between doses for both male and female rats. Relative creatine consumption was higher in the 2.5 g/L (*p* = 0.001), 5 g/L (*p* < 0.001), and 10 g/L (*p* < 0.001) groups compared to the control group. Compared to the 2.5 g/L group, the 5 g/L (*p* = 0.005) and 10 g/L (*p* < 0.001) groups had higher relative creatine consumption. The 10 g/L group consumed more creatine relative to bodyweight than the 5 g/L group (*p* < 0.001). No differences were observed across treatment groups for male or female adipose tissue protein content (ug protein/mg tissue; Table 1). Using rat weights and water consumption data, it is estimated that rats consumed 0.22 g/kg/day (2.5 g/L group), 0.41 g/kg/day (5 g/L group), and 0.79 g/kg/day (10 g/L group) with no difference in relative creatine intake between sexes. The Human Equivalent Dose based on body surface area adjustments [35] suggests that these doses would be equivalent to 2.5, 4.7, and 9.0 g/day in a 7-kg human—normal ranges of CM supplementation.

### 3.2. Creatine Supplementation Increases Mitochondrial Markers in Female Rat Inguinal White Adipose Tissue

**Figure 1:**
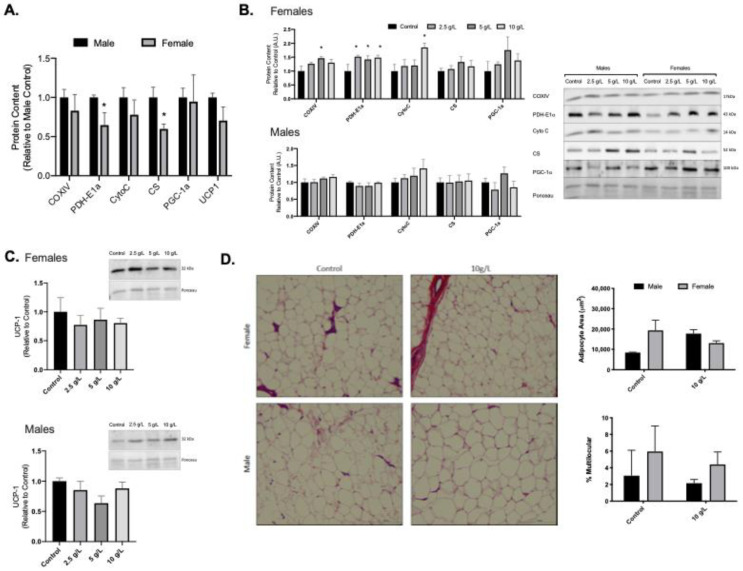
Subcutaneous inguinal white adipose tissue (iWAT) mitochondrial protein content and morphology. (**A**) Quantified Western blot data for iWAT protein content of COXIV, PDH-E1α, Cytochrome C (Cyto C), citrate synthase (CS), PGC-1α, and uncoupling protein-1 (UCP1) for control male and female rats. (**B**) Quantified Western blot data for mitochondrial proteins across creatine supplemented groups and representative Western blot images. (**C**) UCP-1 content in male and female rats across creatine supplemented groups. (**D**). Representative images of H&E stained slides. Scale bar on H&E representative image represents 100 μm. Data were analyzed by ANOVA and are presented as mean ± SEM, * denotes significantly different from control group, *p* < 0.05.

Female rats had lower PDH-E1 alpha and citrate synthase protein content in inguinal subcutaneous adipose tissue when compared to male rats (*p* < 0.05; Figure 1A). Creatine supplementation did not alter mitochondrial protein content in male rat inguinal subcutaneous adipose tissue (Figure 1B). In female rats, creatine supplementation resulted in a higher COVIX (5 g/L), PDH-E1alpha (2.5 g/L, 5 g/L, and 10 g/L), and cytochrome C (10 g/L) content compared to control females (*p* < 0.05; Figure 1B). No differences were observed in UCP1 content in male or female rats (Figure 1C). No differences were observed for male or female adipocyte area or % multilocular in the 10 g/L creatine supplemented group compared to control (Figure 1D).

### 3.3. Creatine Supplementation increases Mitochondrial Markers in Male Rat Gonadal White Adipose Tissue

**Figure 2:**
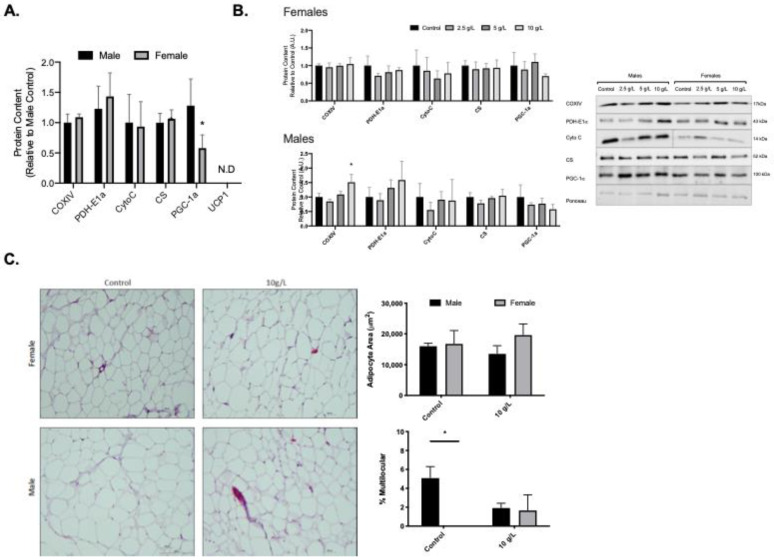
Visceral gonadal white adipose tissue (gWAT) mitochondrial protein content and morphology. (**A**) Quantified Western blot data for gWAT protein content of COXIV, PDH-E1α, Cytochrome C (Cyto C), citrate synthase (CS), PGC-1α, and uncoupling protein-1 (UCP1) for control male and female rats. (**B**) Quantified Western blot data for mitochondrial proteins across creatine supplemented groups and representative Western blot images. (**C**) Representative images of H&E stained slides. Scale bar on H&E representative image represents 100 μm. Data were analyzed by ANOVA and are presented as mean ± SEM, * denotes significantly different from control group, *p* < 0.05.

Female rats had lower PGC-1alpha content in gonadal visceral adipose tissue compared to male rats (*p* < 0.05; Figure 2A). Creatine supplementation did not alter mitochondrial protein content in female rat gonadal visceral adipose tissue (Figure 2B). In male rats, creatine supplementation resulted in a higher COXIV content (10 g/L vs. all other groups, *p* < 0.05) and PDH-E1alpha content (10 g/L vs. control and 2.5 g/L, and 5 g/L vs. 2.5 g/L, *p* < 0.05) (Figure 2B). No differences were observed for male or female adipocyte area (Figure 2C). Control male rats had higher % multilocular adipocytes compared to control females (Figure 2C). No differences in % multilocular adipocytes were observed in the 10 g/L creatine supplemented group compared to control or between sexes (Figure 2C).

### 3.4. Creatine Supplementation does Not increase Mitochondrial Markers Brown Adipose Tissue in either Male or Female Rats

**Figure 3:**
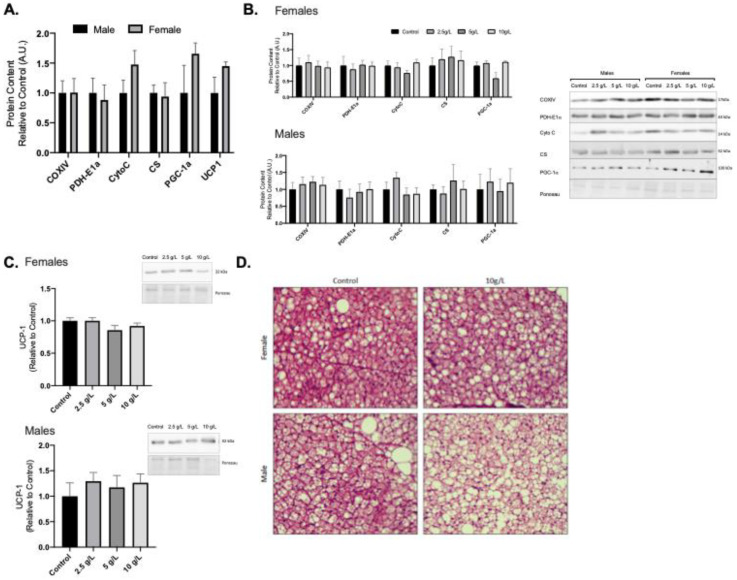
Brown adipose tissue (BAT) mitochondrial protein content and morphology. (**A**) Quantified Western blot data for BAT protein content of COXIV, PDH-E1α, Cytochrome C (Cyto C), citrate synthase (CS), PGC-1α, and uncoupling protein-1 (UCP1) for control male and female rats. (**B**) Quantified Western blot data for mitochondrial proteins across creatine supplemented groups and representative Western blot images. (**C**) UCP-1 content in male and female rats across creatine supplemented groups. (**D**) Representative images of H&E stained slides. Scale bar on H&E representative image represents 100 μm. Data were analyzed by ANOVA and are presented as mean ± SEM, denotes significantly different from control group, *p* < 0.05.

Female rats displayed higher PGC-1alpha content compared to male rats (Figure 3A). Creatine supplementation did not alter mitochondrial protein content in either male or female BAT at any of the supplemented doses (Figure 3B, *p* < 0.05). No differences were observed for UCP1 content in male or female rats across all creatine supplemented groups (Figure 3C). No differences were observed for male or female adipocyte area or % multilocular in the 10 g/L creatine supplemented group compared to control (Figure 3D).

### 3.5. Creatine Supplementation Alters Markers of Creatine Uptake and Metabolism

**Figure 4:**
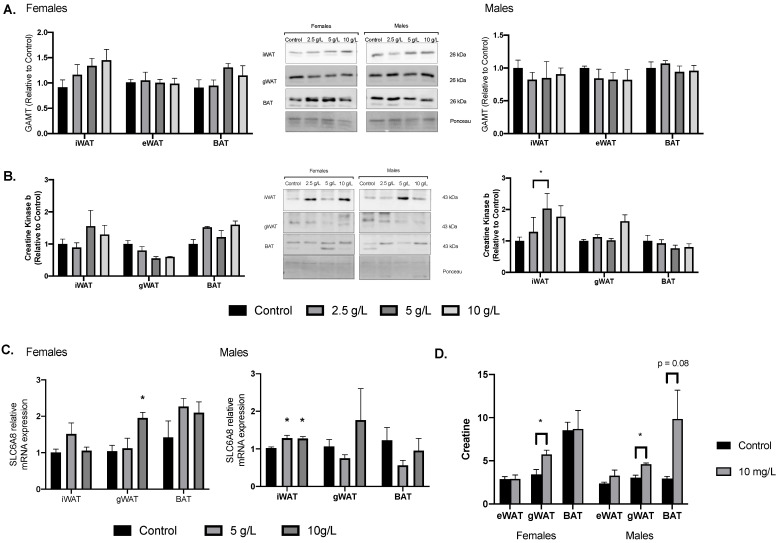
Adipose tissue markers of creatine uptake and metabolism. (**A**) Adipose tissue depot GAMT protein content in female (left) and male (right) rats. (**B**) Creatine kinase B content. (**C**) Slc6A8 mRNA expression. (**D**) Creatine content. Data were analyzed by ANOVA and are presented as mean ± SEM, * denotes significantly different from control group, *p* < 0.05.

Examination of GAMT protein content, a catalyst enzyme responsible for mediating intrinsic creatine synthesis, revealed no differences for GAMT content across any of the creatine dosages or sexes (Figure 4A). CKB was analyzed as a marker of creatine cycling. CKB content was higher in female iWAT at 5 g/L compared to control, this did not reach significance in the 10 g/L group. There were no differences observed in the other depots and no differences observed in the male samples (Figure 4B). As a marker of alterations in creatine transport, *Slc6a8* expression was examined. *Slc6a8* expression was higher in gWAT of female rats treated with 10 g/L creatine compared to controls with no differences in the other adipose depots (Figure 4C). In male rats, 5 and 10 g/L creatine supplementation resulted in higher *Slc6a8* expression in iWAT (Figure 4C). Finally, creatine content was examined in the adipose depots of the 10 g/L groups and compared to the control groups. Creatine content was measured with a commercially available assay kit and was performed as outlined in the assay kit instructions (Biovision, Catalog #K635-100). Creatine content was higher in female and male iWAT within the 10 g/L dose with no differences in the other adipose depots (Figure 4D).

## 4. Discussion

Futile creatine cycling has been identified as a mechanism involved in adipose tissue browning and thermogenesis [14,20,21,22,23,24]. This study examined the potential for creatine supplementation to alter mitochondrial markers in WAT in both male and female Sprague-Dawley rats. Our novel results demonstrate that male and female WAT have a depot specific and sex dependent response to creatine supplementation. Creatine supplementation resulted in an increased creatine content in iWAT in the female and male rats. This was accompanied by increased mitochondrial markers (COXIV, PDH-E1alpha, and cytochrome C) in female rats. No effects on mitochondrial protein markers were observed in male iWAT depots, despite an increase in creatine content. Alternatively, there were no changes in response to creatine supplementation in female rat gWAT, however in male rats, creatine supplementation increased mitochondrial markers (COXIV and PDH-E1alpha) in gWAT. Together, these results highlight creatine supplementation as a potential means to increase WAT mitochondrial content and further highlight the importance of examining sex differences when studying adipose tissue.

Historically, adipose mediated thermogenesis has focused on the role of UCP1, however, it has become apparent that there are UCP1 independent mechanisms that contribute to adipose tissue browning and thermogenesis. Creatine supports energy expenditure in adipocytes and recent work has highlighted a role for futile creatine cycling in adipose tissue thermogenesis [14,19,20,21,22,23,24,36,37]. The potential underlying mechanisms that link creatine to thermogenic respiration have been recently reviewed [8,37]. However, it was not until recently that work has shed some light into the exact underlying mechanisms. Briefly, it has been determined that creatine elicits a substrate cycle of mitochondrial ATP turnover in a sub-stoichiometric fashion [37] and that the recruitment of mitochondrial CKB plays a crucial role in the transfer of phosphate between ATP and creatine [24]. Recently, TNAP was also identified to play an important role [25]. In thermogenic adipocytes, TNAP localizes to mitochondria where it initiates the futile cycling of creatine dephosphorylation and phosphorylation [25]. The potential of dietary creatine supplementation to stimulate adipose tissue browning has yet to be fully investigated. Kazak et al. examined creatine supplementation in Adipo-Gatm KO mice and found that supplementation rescued impaired adrenergic thermogenesis in these mice [22]. Here, we demonstrated that 8 weeks of creatine supplementation results in increased mitochondrial markers in WAT depots with no change in UCP1 content. This is an important and novel finding as it suggests that dietary creatine may be a means to improve adipose tissue health and possibly increase thermogenesis, independent of UCP1. One limitation of this work is the lack of functional outcomes at the tissue and whole-body level. Future work should determine if these increases in mitochondrial protein content result in enhanced mitochondria respiration and in turn if this enhances whole-body energy expenditure. While there were no differences in body mass amongst groups at the end of the intervention, it is possible that longer term supplementation or supplementation in conjunction with exercise training may result in significant reductions in body mass.

Interestingly, the observed increases in mitochondrial markers were sex- and depot- specific. Our findings show that in female rats, 8 weeks of creatine supplementation resulted in mitochondrial adaptations in inguinal subcutaneous WAT with no changes in the visceral WAT, while the opposite was true for male rats. Much information about adipose tissue depots specific differences has accumulated over the past few decades. It is known that the type of adipose tissue and the location in which it accumulates is important with regard to disease risk. For example, the accumulation of visceral WAT is associated with an increased risk of insulin resistance, type 2 diabetes, dyslipidemia, and atherosclerosis [38,39], while subcutaneous WAT is associated with higher insulin sensitivity and a reduced risk of type 2 diabetes [40,41,42]. The underlying mechanisms for the varying responses and metabolic effects of subcutaneous and visceral fat are most likely due to unique properties within the depots. Subcutaneous and visceral adipocytes develop from different progenitor cell lines, differentiate at varying rates, and can develop unique gene expression profiles [43,44]. For example, the expression of PRDM16, a transcription coregulatory protein responsible for adipose tissue browning, is much higher in subcutaneous WAT compared to visceral WAT [45]. It is known that beige cells are found interspersed in the WAT of humans and rodents [46,47,48], and the browning occurs predominantly in subcutaneous WAT. These differences between adipose tissue depots could account for the diverse responses to dietary creatine supplementation in our study. However, further investigation is needed to fully determine the molecular mechanisms underlying the creatine-induced mitochondrial changes and how this regulation is specific to each adipose tissue depot in both sexes. Future studies should explore differences in creatine transporter content and uptake across depots and sexes.

Animal models have demonstrated that sex and sex hormones can influence adipose tissue development, adipogenesis, gene expression profiles regulating insulin resistance and lipolysis, as well as the inflammatory tone and remodeling responses to obesity [49]. It is possible that the observed differences in response to creatine supplementation are due to circulating sex hormones. Previous work has shown that subcutaneous WAT has a higher concentration of estrogen receptors and progesterone receptors compared to androgen receptors in females. In contrast, visceral WAT has a higher concentration of androgen receptors [49,50]. This differential expression of sex hormone receptors could have influenced the response to creatine supplementation observed here in female and male rats. Interestingly, in differentiated 3T3L1 adipocytes, estradiol stimulated the specific activity of creatine kinase [51,52]. This highlights the differential response in adipose depots across sexes and together with our results sets the groundwork for future work in the area.

In the current study, mRNA analysis was conducted to examine if creatine supplementation had an effect on the expression of the creatine transporter (*Slc6a8)*. Differences were demonstrated in a sex- and depot-specific manner. Females exhibited increases of the *Slc6a8* gene within the gWAT adipose depot, whereas males exhibited this increase in the iWAT depot. This finding is compelling as it contrasts our other findings, which demonstrated that females experienced mitochondrial protein increases in iWAT and males in the gWAT. As explained previously, subcutaneous iWAT has higher concentrations of estrogen receptors in females, whereas visceral gWAT has higher concentrations of androgen receptors. Therefore, it is possible this finding may be explained as an adaptive physiological response; the *Slc6a8* gene may be upregulated in female gWAT depots and male iWAT depots in an attempt to produce equal physiological responses among different adipose depots within the same rat, however, this is purely speculative.

The current study provides new information demonstrating the potential of dietary creatine supplementation on improving WAT health and increasing mitochondrial markers. Of note are the novel sex- and depot- specific responses to creatine supplementation. These highlight the importance of examining sex-differences in adipose tissue. Future work should explore the depot- and sex- specific responses to creatine. Importantly, the results presented here highlight the efficacy of creatine supplementation to increase mitochondrial proteins and highlight the potential as a preventative or therapeutic treatment for obesity and related metabolic diseases.

## Figures and Tables

**Table 1:** Animal and adipose depot characteristics.

Animal Characteristics				
Creatine (g·L^−1^)	0	2.5 ^$^	5 ^&^	10
End point body mass (g)				
Males	587.8 ± 9.1	602.8 ± 14.1	610.3 ± 25.3	584.5 ±23.0
Females	376.8 ± 13.5 *	416.3 ±13.5 *	397.0 ± 7.1 *	404.8 ± 28.3 *
Food Intake (g·day^−1^)				
Males	24.4 ± 0.3	24.3 ± 0.9	23.7 ± 1.7	23.8 ± 1.3
Females	17.7 ± 1.2 *	19.0 ± 0.7 *	18.3 ± 0.1 *	18.75 ± 1.2 *
Water Intake (mL·day^−1^)				
Males	57.9 ± 14.0	44.9 ± 8.7	36.1 ± 2.2 ^#^	43.5 ± 1.0
Females	34.7 ± 4.6 *	35.8 ± 5.4 *	35.4 ± 1.9 *	27.9 ± 1.3 *
Gonadal Adipose Tissue Protein Content (ug protein/mg tissue)				
Males	19.4 ± 1.3	19.4 ± 1.2	18.2 ± 0.3	19.1 ± 1.3
Females	19.5 ± 1.0	18.0 ± 1.1	18.1 ± 0.3	18.1 ± 0.7
Inguinal Adipose Tissue Protein Content (ug protein/mg tissue)				
Males	28.2 ± 1.2	27.5 ± 3.9	33.9 ± 6.0	29.0 ± 0.9
Females	29.0 ± 4.6	25.1 ± 1.0	29.8 ± 1.7	29.9 ± 0.9
Interscapular Brown Adipose Tissue Protein Content (ug protein/mg tissue)				
Males	93.4 ± 5.4	111.6 ± 12.2	99.6 ± 9.4	107.2 ± 10.8
Females	112.0 ± 9.4	106.3 ± 12.2	117.2 ± 7.6	120.9 ± 3.1

End point body mass, total food intake, water intake, and creatine intake. Data are presented as means ± SD. * *p* < 0.05 compared to males in the same treatment group. ^#^ *p* < 0.05 compared to control of same sex. ^$^ *p* < 0.05 compared to 2.5 g/L group of same sex. ^&^ *p* < 0.05 compared to 5 g/L group of same sex.

## Data Availability

Data available upon request.
